# Improved Mass Spectrometry Assay For Plasma Hepcidin: Detection and Characterization of a Novel Hepcidin Isoform

**DOI:** 10.1371/journal.pone.0075518

**Published:** 2013-10-04

**Authors:** Coby M. M. Laarakkers, Erwin T. Wiegerinck, Siem Klaver, Maria Kolodziejczyk, Hendrik Gille, Andreas M. Hohlbaum, Harold Tjalsma, Dorine W. Swinkels

**Affiliations:** 1 Laboratory of Genetic, Endocrine and Metabolic Diseases, Department of Laboratory Medicine, Radboud University Medical Centre, Nijmegen, The Netherlands; 2 Hepcidinanalysis.com, Nijmegen, The Netherlands; 3 Pieris AG, Freising, Germany; University of Warwick – Medical School, United Kingdom

## Abstract

Mass spectrometry (MS)-based assays for the quantification of the iron regulatory hormone hepcidin are pivotal to discriminate between the bioactive 25-amino acid form that can effectively block the sole iron transporter ferroportin and other naturally occurring smaller isoforms without a known role in iron metabolism. Here we describe the design, validation and use of a novel stable hepcidin-25^+40^ isotope as internal standard for quantification. Importantly, the relative large mass shift of 40 Da makes this isotope also suitable for easy-to-use medium resolution linear time-of-flight (TOF) platforms. As expected, implementation of hepcidin-25^+40^ as internal standard in our weak cation exchange (WCX) TOF MS method yielded very low inter/intra run coefficients of variation. Surprisingly, however, in samples from kidney disease patients, we detected a novel peak (m/z 2673.9) with low intensity that could be identified as hepcidin-24 and had previously remained unnoticed due to peak interference with the formerly used internal standard. Using a cell-based bioassay it was shown that synthetic hepcidin-24 was, like the -22 and -20 isoforms, a significantly less potent inducer of ferroportin degradation than hepcidin-25. During prolonged storage of plasma at room temperature, we observed that a decrease in plasma hepcidin-25 was paralleled by an increase in the levels of the hepcidin-24, -22 and -20 isoforms. This provides first evidence that all determinants for the conversion of hepcidin-25 to smaller inactive isoforms are present in the circulation, which may contribute to the functional suppression of hepcidin-25, that is significantly elevated in patients with renal impairment. The present update of our hepcidin TOF MS assay together with improved insights in the source and preparation of the internal standard, and sample stability will further improve our understanding of circulating hepcidin and pave the way towards further optimization and standardization of plasma hepcidin assays.

## Introduction

The peptide hormone hepcidin plays a central role in regulating dietary iron absorption and body iron distribution. Many human diseases are associated with alterations in hepcidin concentrations. The measurement of hepcidin in biological fluids is therefore a promising tool in the diagnosis and management of medical conditions in which iron metabolism is affected [1]. Hepcidin is a 25 amino-acid peptide hormone that is predominantly produced by hepatocytes and regulates systemic iron homeostasis. Under physiological conditions N−terminal truncated hepcidin−20 and −22 peptides have been observed in the urine, but not or at low concentrations, in plasma [2]–[7]. These smaller hepcidin isoforms mostly occur in plasma in diseases that are associated with significantly increased hepcidin concentrations, such as sepsis and kidney failure [6], [8]–[10]. Much is still unknown about the origin of the smaller isoforms. Data suggest that a calcium-independent tissue activity present in pancreas extracts might be responsible for the systemic N-terminal truncation of hepcidin-25 to hepcidin-22, and that dipeptidylpeptidase 4 is involved in the processing of hepcidin-22 into hepcidin-20 [11], [12]. It is however unknown whether hepcidin isoforms can also be the result of ex-vivo processing. Thus far, we and others measured hepcidin using a Weak Cation Exchange Time−of−Flight Mass Spectrometry (WCX−TOF MS) method, with the synthetic hepcidin−24 (desAsp-hepcidin-25) analogue spiked into the sample as an internal standard for quantification [5], [6], [10]. This hepcidin analogue was chosen because these assays were run on low/medium resolution platforms such as surface-enhanced and matrix-assisted laser desorption/ionization (SELDI/MALDI) TOF MS platforms that need a relatively large mass difference to separately detect two peptide peaks. Despite the slightly different biochemical characteristics, the binding of desAsp-hepcidin and hepcidin-25 to WCX beads were similar [5], but different for IMAC-Cu^2+^ chips as applied in SELDI-TOF measurements (hepcidin-24/hepcidin-25 ratio’s 0.71 and 0.93, as observed by Swinkels et al. [5] and Campostrini et al. [10], respectively). Importantly, the implementation of this internal standard to correct for inter-assay variation proved to be very useful and has successfully detected physiologic and pathologic changes in serum hepcidin in patients with various disorders of iron homeostasis [1], [6], [9], [13]–[18]. However, the exact impact on the use of desAsp-hepcidin as an internal standard on the accurate quantification of hepcidin remained unclear, especially in disorders with increased concentrations of the smaller hepcidin isoforms.

Reliable quantitative hepcidin assays have been developed on mass spectrometry and immunochemical platforms. The mass spectrometry based methods can roughly be divided in those run on low/medium resolution platforms [5], [6], [10], [19] and those run on high resolution platforms [20]–[25]. Most hepcidin assays on the latter platforms report the use of internal standards that comprise stable hepcidin isotopes causing mass shifts that vary between 8 and 14 Da [20]–[25].

At present there are considerable differences in hepcidin measurements using the various methodologies. Our two published international hepcidin round robins (distribution of identical samples to allow inter-laboratory assay comparisons) revealed i) generally high correlations between the participating methods and ii) similar between-sample and analytical variation of most methods [25], [26]. However, absolute hepcidin concentrations differed widely between the assays. These discrepancies might be attributed to differences in the values that laboratories and companies assign to the internal and external standards used for the different methods, to impurities in these standards or to loss of the standard during storage, e.g. by aggregation [27] or differential adsorption of the synthetic hepcidins to tubes or other surfaces of laboratory materials. Hence, to bridge this gap there is a need for improved insights on accurate value assignment and optimal handling of synthetic hepcidin standards to allow the formulation of recommendations on this point.

In this article, we describe an update of our assay by replacing desAsp-hepcidin (hepcidin-24) as internal standard with a newly designed stable heavy isotope hepcidin-25 that has an increased molecular weight of 40 Da compared to native hepcidin-25. Notably, this relative large mass shift makes this isotope suitable for both low/medium resolution linear time-of-flight (TOF) platforms as well as high resolution mass spectrometry instruments. We validated this updated assay by assessing its reproducibility and its clinical relevance by measuring samples from various patients groups. We moreover investigated storage stability of hepcidin-25 and its smaller isoforms in samples derived from different disease entities under various conditions (anticoagulant, storage temperature and time). An unexpected finding concerned the identification of hepcidin-24 as a novel natural isoform in samples from kidney disease patients. We quantified the bioactivity of this hepcidin-24 isoform relative to bioactive hepcidin-25, and the -22 and -20 isoforms by assessing its ability to internalize and degrade ferroportin in a cell−based assay. Finally, we investigated the effect of the source and handling of different internal standards that are currently in use on the value assignment of the hepcidin results. These data aid in the understanding of circulating hepcidin-25 and its isoforms and the further optimization and standardization of plasma hepcidin assays throughout the world.

## Materials and Methods

### Sample Collection and Human Specimens

#### Ethics Statement

Use of serum and plasma from patients and healthy donors was approved by the Medical Ethical Committee of the district Arnhem-Nijmegen (The Netherlands) as its use in our studies conforms to the code for proper secondary use (“goed gebruik”) of human tissue in the Netherlands and the declaration of Helsinki, respectively. Written informed consent was obtained from all healthy donors.

#### Blank plasma and serum

Hepcidin-25 blank serum was derived in April 2010 from an iron-depleted patient with juvenile hemochromatosis [28]; Hepcidin-25 blank heparin-plasma was obtained from a iron-deficient female donor in September 2012 and from the iron-depleted patient with juvenile hemochromatosis in February 2013.

#### Plasma from intensive care and Nephrology patients

The samples of patients from the intensive care unit (IC) and the nephrology department concerned leftovers from routine blood draws. Samples were used for measurement of hepcidin between 4–7 hrs after blood drawing and/or pooled and/or stored in aliquots at various temperatures for prolonged periods of time. EDTA (n = 10) and/or heparin plasma (n = 20) samples from IC patients were obtained in plastic tubes in October-November 2012. Heparin and EDTA samples of patients on hemodialysis (HD) were derived from aliquots (stored at −80°C) from a previous study collected between August and October 2010 [16]. For use in specific experiments, plasma pools were generated by collection of leftover samples within 24–48 hrs (at 4°C) after routine blood draw: i) heparin plasma from 30 different IC patients was collected in November-2011, ii) heparine plasma from 20 different nephrology patients was collected in November 2011 to obtain 2 different pools.

#### Serum and plasma from healthy controls

Serum samples from healthy controls that were used in this study concerned leftover aliquots (stored at −80°C) that were collected in February 2011 [18]. In addition, citrate plasma, heparin plasma, EDTA plasma and serum for stability studies was drawn in the afternoon from healthy volunteers (n = 5), known for having fasted morning hepcidin levels above the lower limit of detection (>0.5 nM) in September 2012. Fresh samples were measured within 2–4 hrs hours after blood drawing and stored in aliquots at various temperatures for prolonged incubation.

#### Quality Control (QC) samples

Heparin plasma QC samples (n = 2), high and low (HiQC2011 and LoQC2011), were obtained in November 2011 by pooling sample leftover samples from IC patients and iron-depleted HFE-hemochromatosis patients, respectively. Serum QC samples (n = 2), high and low (HiQC2009 and LoQC2009), were obtained in April 2009 from 2 blood donors from the local blood donation centre. To obtain the high QC sample, serum of one of the donors with a native hepcidin concentration <1 nM was spiked with synthetic hepcidin-25 from Peptide International (PI, Louisville, KY, USA) to a concentration of 9 nM.

### Artificial Samples

Synthetic-hepcidin (100 nM in 20% acetonitril) is spiked into blank plasma or serum.

### MALDI-TOF MS

WCX-TOF MS was performed between October 2011 and March 2013 as described previously by a combination of WCX bead-based hepcidin enrichment followed by TOF-MS [6]. As internal standards for quantification were used: i) synthetic hepcidin-24 (custom made Peptide International, 2673.9 Da; [5]); ii) novel custom made heavy hepcidin-25^+40^ (see below, Table 1). Mass-to-charge (*m/z*) spectra were generated by using MALDI-TOF MS (Microflex LT, Bruker Daltonics). Spectra were analyzed by using Bruker Daltonics FlexAnalysis software. A detailed protocol of this method is described in [6]. Total hepcidin concentration was defined as the sum of hepcidin-25, -24, -22, and -20 concentrations.

**Table 1:** Characteristics of commercial hepcidin peptides used.

Hepcidin peptide	Commercial source	Product no	Lot number	MW (Da)	pI	Peptide content	Purity
Heavy hepcidin-25	PI*	n.a.	221-006221	2829.4†	8.22	100%∧	>97.8%
Hepcidin-25	PI*B	PLP-4392-s	6103103005438	2789.4	8.22	100%∧68.6%	>98%>95%
Hepcidin-24	PI*	n.a.	740-609191	2673.9	8.51	100%∧	96.3%
Hepcidin-22#	PI	PLP-3776 PI	001055V	2436.1	8.53	74.5%	96.8%
Hepcidin-20#	PI	PLP-3777-PI	001041V	2191.8	8.53	67.6%	96.1%

* catalog product of Peptide International but manufactured by Peptide Institute (Osaka, Japan);

∧ vialed at 100% peptide content;

† due to an isotope content of 98% the actual mass is 1 Da less than the theoretical mass of this peptide.

# delivered as 0.10 mg per vial; personal communication revealed that ≈ 0.11 mg was pipetted in each vial. PI, Peptide International (Louisville, KY, USA); B, Bachem LTD (St. Helens, UK); n.a., not applicable.

### Novel Internal Standard Heavy Hepcidin-25^+40^

Heavy hepcidin-25^+40^ (hepcidin-25^+40 ^Da; DTHF(^13^C_9_,^15^N)P(^13^C_5_,^15^N)I(^13^C_6_,^15^N)CI(^13^C_6_,^15^N) F(^13^C_9_,^15^N)CCG(^15^N)CCHRSKCGMCCKT disulfide bridged) was obtained from Peptides International (Table 1). Note that due to an isotope content of 98% the actual mass is 1 Da less than the theoretical mass of this peptide. Freeze dried hepcidin-25^+40^ was dissolved in H_2_O in accordance with the manual. An 0.1 nmol/L solution of hepcidin-25^+40^ was aliquoted in 12 µL volumes in 200 µL polypropylene tubes and stored at −80°C until use.

### Other Synthetic Hepcidin Peptides

In certain experiments, hepcidin-25 from Peptide International and Bachem LTD, were used (Table 1). Hepcidin-24 (custom), hepcidin-22 and hepcidin-20 were purchased from Peptide International. Confusingly, both Peptides International and Bachem differed in relative peptide content of the vials. Bachem provided the gross amount of hepcidin on the vial that (according to the package insert) consisted of 68.6% hepcidin-25 (as assessed by amino-acid analysis: the remaining being salts and water). In addition, peptide contents of catalog products of Peptides International manufactured by Peptides International itself (USA) were lower (65–75%) than those manufactured by Peptide Institute in Osaka, Japan (Table 1). According to manufacturers protocol both peptides had disulfide bonds between Cys1–Cys8, Cys2–Cys7, Cys3–Cys6, and Cys4–Cys5. Purity assessed by HPLC was >96.1–98% for Peptide International hepcidin (-isoforms) and >95% for Bachem hepcidin-25. Both companies did not determine the nature of the impurities, but in the case of peptides with multiple disulphide bridges as with hepcidin, they may concern misfolded peptides.

### Pre-treatment with Anti-hepcidin Molecules

For *in vitro* immune-depletion studies, we used the Anticalin PRS-080, a highly specific and potent hepcidin antagonist exhibiting an affinity of 50 pM for hepcidin-25 and its amino−terminal truncated versions including hepcidin-20 (Pieris AG, Freising, Germany; http://www.eurocalin-fp7.eu/; [29]). Anticalins are engineered human lipocalins where the natural ligand binding pocket is re-designed to bind therapeutically relevant targets in a monovalent fashion with a molar ratio of 1∶1. This has also been experimentally confirmed for PRS-080 and other Anticalins through X-ray analysis of crystal structures of Anticalins in complex with their target ([30] and unpublished results). The exquisite binding specificity and selectivity of the Anticalin was investigated in detail with various methods including surface plasmon resonance in which the Anticalin did not exhibit any measurable affinity towards a range of structurally related and non related targets ([29]; and unpublished results).

Importantly, use of PRS-080 in a non-human primate model did effectively increase serum iron levels showing its ability to bind and inactivate hepcidin *in vivo* [29]. In the *in vitro* experiments described here, plasma and serum samples were incubated with known concentrations of PRS-080 (diluted in PBS) for half an hour at room temperature (RT), before employment in the WCX-TOF MS assay. Concentration of PRS-080 was determined by the UV280 method with a coefficient of variation of about 10% [31].

### Hepcidin Bioactivity Assay

A stable cell line engineered for inducible expression of green fluorescent protein (GFP)-fused human ferroportin was used as a test system to determine hepcidin-24 activity relative to hepcidin-25, 22 and 20. In this cell system, ferroportin internalization and degradation provided a means to quantify hepcidin activity. The assay was implemented basically as described by Nemeth et al. [32], [33] except that fluorescence was quantified in triplicate wells on a microplate reader. To determine EC_50_ values for hepcidin-25 and hepcidin-24, a curve was fitted by nonlinear regression with a four parameter logistic equation using Prism v5 (GraphPad).

### Hepcidin Stability Experiments

Using the novel heavy hepcidin-25 internal standard, we assessed changes in hepcidin-25 and the smaller isoforms in various samples containing various anticoagulants *ex vivo* at room temperature (RT), 4°C, −20°C and −80°C. More specifically, changes in hepcidin concentrations relative to those found in freshly collected samples were determined i) at storage at RT and 4°C on day 0 (fresh samples), 1 (14–22 hrs), 2, 3, and 7 days after sampling/storage, ii) at −20°C after 1 week, and after 1, 4 and 6 months of storage and iii) at −80°C at after 6 months of storage. We also assessed the effect of the addition of protease inhibitors on the stability of hepcidin concentrations of heparin plasma of IC patients at RT during a 1 week storage. To this end, before storage we used 1 tablet of protease inhibitor (Complete, Mini, Roche Diagnostics, Mannheim, Germany) solved in 200 µL PBS, of which 20 µL was added to 1 ml plasma. Finally, we used our QC charts to assess the stability of hepcidin concentrations in heparin plasma and serum QC aliquots stored at −80°C for 2 years.

## Results and Discussion

### Design of a Heavy Hepcidin-25 Isotope for Linear TOF MS Application

Over the last years, we and others have quantified hepcidin-25 using affinity-enrichment followed by a linear TOF MS method with synthetic hepcidin−24 (desAsp-hepcidin-25) spiked into the sample as an internal standard for quantification [5], [6], [10]. The mass difference of 115.5 Da between synthetic hepcidin-24 and native hepcidin-25 makes resolution of both peaks highly convenient on low/medium resolution SELDI−/MALDI-TOF MS platforms. However, due to the slightly different biochemical characteristics (Table 1), the behavior of both peptides appeared different in the IMAC-Cu^2+^ SELDI-TOF approach, which motivated us to set-up hepcidin measurements using a weak cation exchange (WCX-)MALDI approach in which both peptides performed exactly the same. An important incentive of the present study was to design a broadly applicable heavy hepcidin-25 peptide that can be used as internal standard in different assay formats on various mass spectrometry platforms as this might contribute to the harmonization of the different hepcidin assays throughout the world [25]. Most assays run on high resolution MS platforms employ hepcidin-25 standards that cause a mass shift of hepcidin-25 between +8 and +14 Da [20]–[25]. As the methionine residue in hepcidin molecules is prone to oxidation [5](+16 Da), these relatively small mass shift internal standards would not be adequate on low/medium resolution platforms (SELDI/MALDI-TOF MS) due to peak interference between the internal standard and hepcidin-25-O_2_. Therefore, we designed a heavy hepcidin-25 isotope with a relative large mass shift of +40 Da (molecular weight: 2829.4 Da; see Materials & Methods) because at this position we did not observe other interfering peaks in the WCX-TOF MS spectra of different patient populations (data not shown). The utility of the +40 Da hepcidin internal standard should be verified in high resolution MS platforms as well as in low/medium resolution TOF MS-based assays that employ other peptide enrichment approaches. These studies are foreseen to be part of a 3^rd^ Round Robin for serum hepcidin assays, that we will initiate in 2014.

Using hepcidin-25^+40^ as a new internal standard, we performed validation experiments on different aspects of the WCX-TOF MS assay as outlined below.

### Preparation of the Internal Standard and Effect on Hepcidin-25 Value Assignment

**Figure 1:**
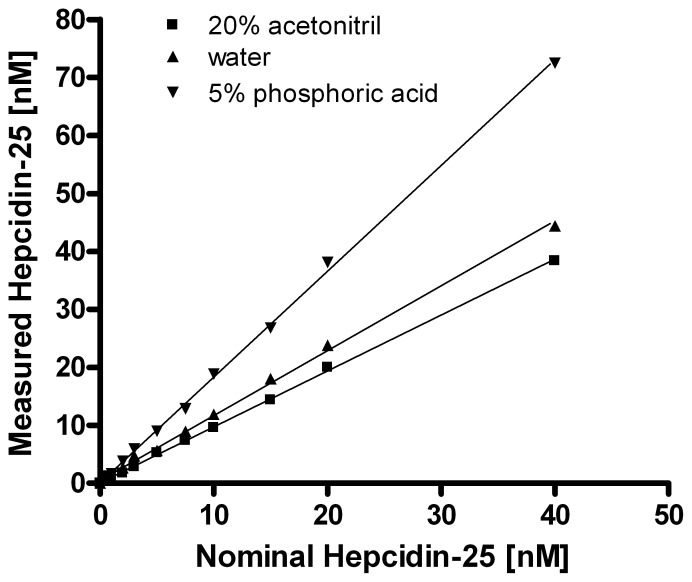
Effect on the solvent of hepcidin-25^+40^ on hepcidin-25 linearity and value assignment. Hepcidin-25^+40^ was dissolved in 20% acetonitril (acn), 5% phosphoric acid or water and used as internal standards in blank serum samples containing end concentrations of 0, 0.5,1, 2, 3, 5, 7.5, 10, 15, 20 and 40 nM synthetic hepcidin-25 purchased from Peptides International. Linearity of measured hepcidin-25 was determined by the formulas: Y = 1.822X+0.097 (5% phosphoric acid); Y = 1.119X+0.494 (water); Y = 0.964X+0.069 (20% acn).

As quantification of native hepcidin in a sample is related to the known concentration of the spiked internal standard, the absolute levels of measured hepcidin are highly dependent on the value assignment of the used internal standard. One determining factor concerns proper dissolvent of the lyophilized peptide and whether or not a substantial amount of the internal standard is lost by aggregation or sticking to laboratory plastics during its preparation. Therefore, we compared the effects of three different solvents to prepare hepcidin-25^+40^ stock solutions on the measured hepcidin-25 concentration of a series of spiked blank serum samples to which known hepcidin-25 concentrations were added. These analyses clearly showed that significantly higher hepcidin-25 concentrations were measured when hepcidin-25^+40^ was dissolved in 5% phosphoric acid, compared to 20% acetonitril and water as dissolvent’s (Figure 1). This finding implicates that use of 5% phosphoric acid leads to loss of about 50% of the peptide, while this seems to be the case for about 15% of the peptide when water is used. As this loss is not corrected for in the value assignment of the internal standard and the native hepcidin peak is related to the intensity of this standard peak (which is too low for its concentration), this results in measured values that are false high. Based on these observations, we defined 20% acetonitril as the best solvent to prepare stock solutions of the internal standard as this yielded a near 1∶1 ratio between the measured hepcidin concentration and the nominal concentration (added concentration) hepcidin (dissolved in 20% acetonitril) in the measured samples. Therefore, 20% acetonitrile was implemented as solvent for the internal standard in our standard operating procedure, which was used throughout the remainder of this study. Notably, such differences in the preparation of internal standards could very well explain in part the high level of variation in absolute hepcidin levels measured by different assays throughout the world [25], [26].

### Quantification of Hepcidin Isoforms using Heavy Hepcidin-25^+40^as Internal Standard

**Figure 2:**
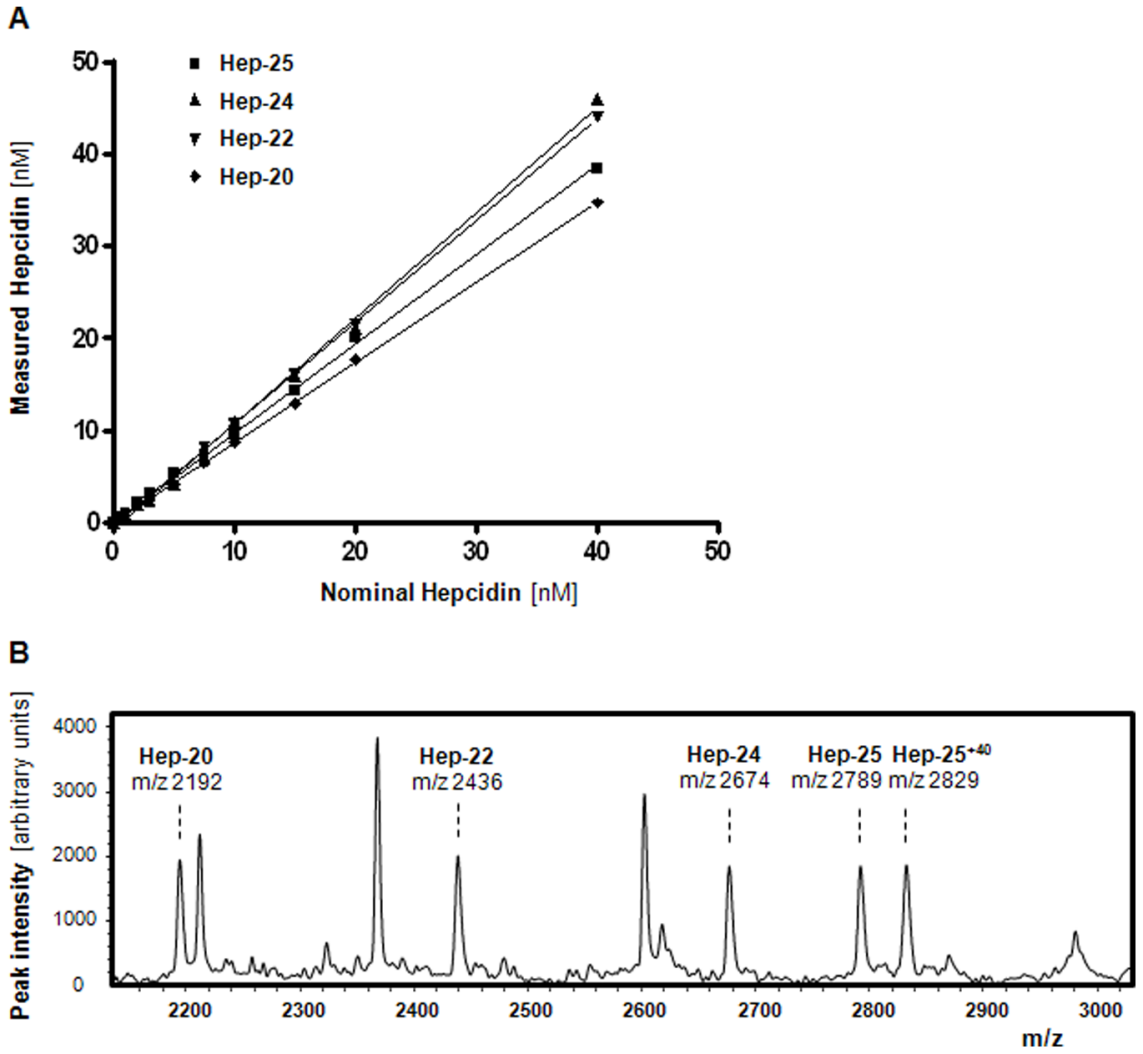
Quantification of hepcidin isoforms using hepcidin-25^+40^ as internal standard. **A.** Linearity (range 0–40 nM) for hepcidin-25, hepcidin-24, hepcidin-22, and hepcidin-20 as determined by hepcidin-25^+40^ as internal standard. Linearity curves are assessed in different runs. Blank serum (hep-24, hep-20 and hep-25) or heparin plasma (used for hep-22 as serum yields an interfering peak near the position of this isoform) was used as matrix for the addition of the synthetic hepcidin isoforms (PI) to end concentrations of 0, 0.5,1, 2, 3, 5, 7.5, 10, 15, 20 and 40 nM. Since there is a small interfering peak at 2191.8 Da in blank serum, the linearity curve of hepcidin-20 was corrected for the base line hepcidin-20 peak (data not shown). Description of the lines: hepcidin-25, Y = 0.964X+0.064 (R^2^ = 0.9950); hepcidin-24, Y = 1.145X−0.767 (R^2^ = 0.9975); hepcidin-22, Y = 1.100X−0.197 (R^2^ = 0.9998); hepcidin-20, Y = 0.867X+0.055 (R^2^ = 0.9998). **B.** WCX-TOF MS profile of blank plasma that was spiked with 10 nM of each of the synthetic hepcidin-20, -22, -24, -25, and -25^+40^ peptides, which illustrates that all these hepcidin analogues have the same WCX-binding characteristics and flight behavior during TOF MS.

To assess whether hepcidin isoforms forms can also be accurately measured using the new internal standard, hepcidin-25^+40^ was spiked as internal standard into blank serum or heparin plasma samples to which synthetic hepcidin-24, -22 and -20 was added in known concentrations. As shown in Figure 2A, this approached showed good linearity in measurements of all three tested hepcidin isoforms, which was comparable to that of hepcidin-25. The small differences in the measured values of the different isoforms remained within the intra-run variation of the assay (Table 2). WCX-binding characteristics and flight behavior during TOF MS was assessed by spiking 10 nM of each of the different synthetic hepcidin peptides to the same blank serum prior to WCX-TOF MS. Figure 2B shows similar peak intensities of all hepcidin peptides, which confirms that hepcidin-25^+40^ internal standard is also an appropriate internal standard to quantification hepcidin-24, -22, and -20 isoforms in our assay.

**Table 2:** Reproducibility of the measurement of hepcidin-25 and its isoforms of the improved hepcidin assay with the novel hepcidin-25^+40^ as internal standard.*

	Intra-run (n = 8)		Inter-run (n = 8)	
Hepcidin form	Level (nM)	CV(%)	Level (nM)	CV %
Hepcidin-25	2.6	2.7	2.6	8.3
	3.5	3.3	10.5	4.6
	7.4	3.5		
	10.3	2.1		
Hepcidin-24	1.4	8.2	2.3	16.8
	2.3	4.0		
	2.4	2.9		
Hepcidin-22	1.4	8.6	1.4	11.6
	1.7	7.8		
Hepcidin-20	2.3	7.6	2.5	13.7
	4.9	4.2		
	5.8	6.0		

* Heparin samples used for intra-run experiments are the high and low QC samples and samples from nephrology patients (n = 2). Heparin samples used for inter-run experiments are the high and low QC samples.

### Effect of Different Plasma Matrices on Measured Hepcidin-25 Concentration

**Figure 3:**
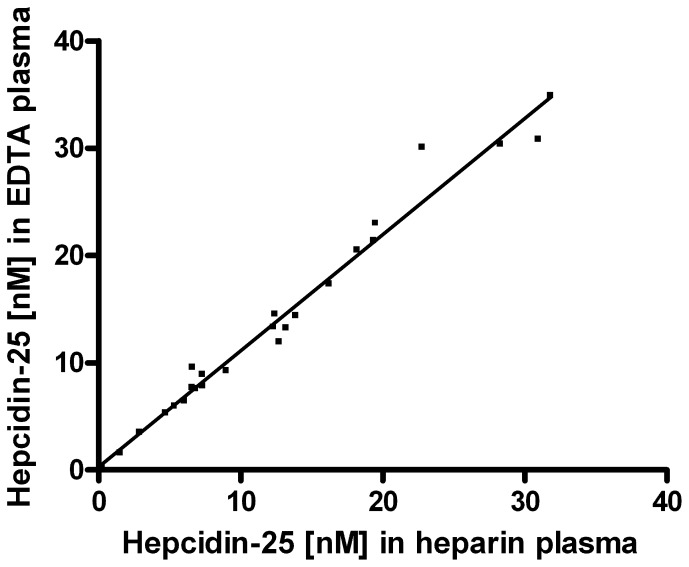
Comparison of hepcidin-25 concentrations measured in EDTA plasma and heparin plasma samples from healthy controls (n = 5), hemodialysis (HD) patients (n = 10) and intensive care (IC) patients (n = 10). Description of the line: Y = 1.083X+0.274 (R^2^ = 0.9770).

To assess the effect of different plasma collection protocols on the measured hepcidin-25 concentration, hepcidin-25^+40^ was added to heparin plasma, EDTA plasma and citrate plasma that was simultaneously collected from the same patients or volunteers and frozen at −80°C until measurements. These comparisons showed good correspondence between hepcidin-25 levels in heparin plasma and citrate plasma (data not shown). However, we measured slightly higher hepcidin-25 concentrations in EDTA plasma compared to heparin plasma (Figure 3) and citrate (not shown). We have currently no clear explanation for this difference. Thus, to properly compare hepcidin data obtained within a study an identical plasma matrix should be used.

### Identification of Hepcidin-24 as Native Hepcidin Isoform

**Figure 4:**
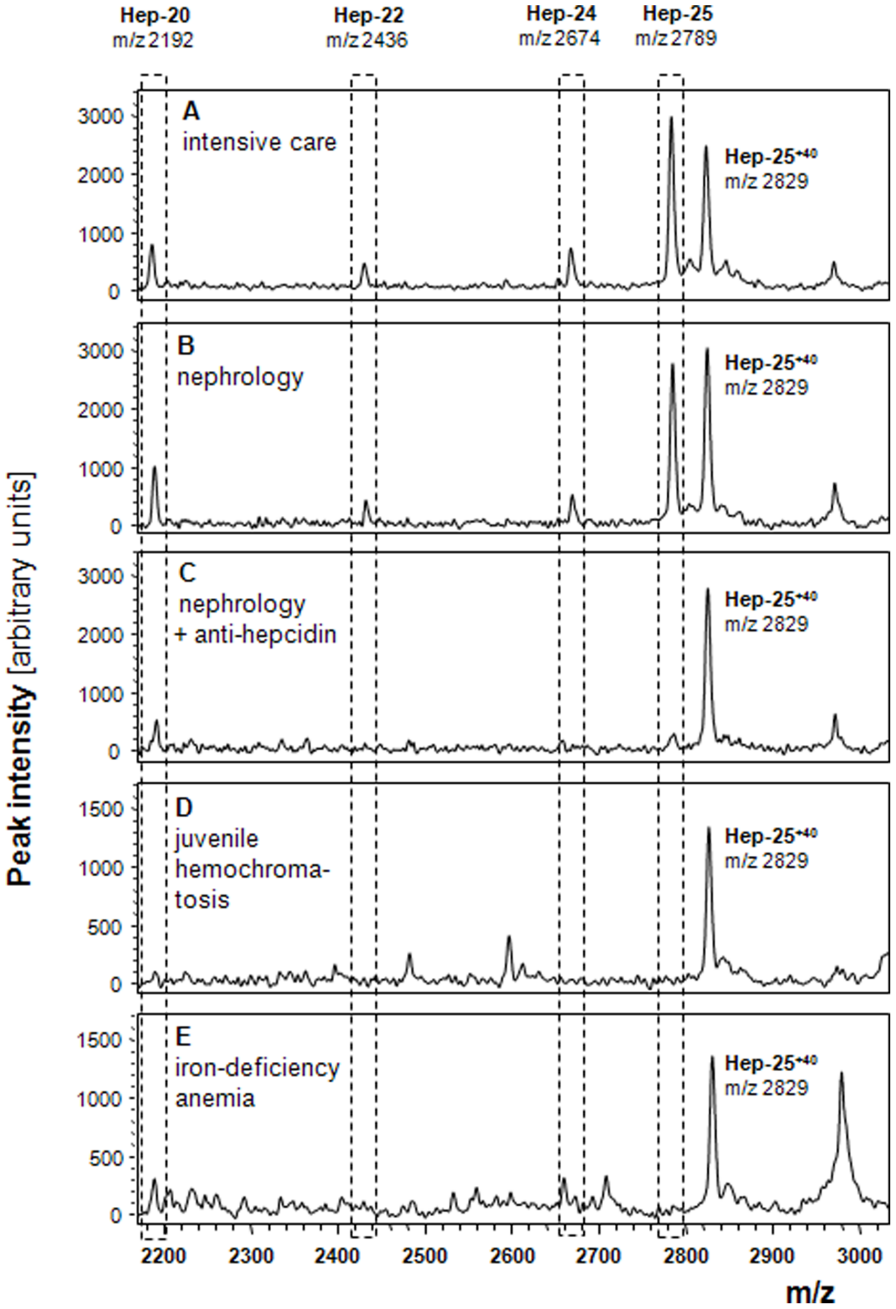
WCX-TOF MS profiles of sample pools of patients with presumed hepcidin isoforms. Panel **A**, peptide profile of a heparin plasma pool of IC patients; Panel **B/C**, peptide profile of heparin plasma pool of nephrology patients that were untreated (**B**) or pre-incubated with 1 molar excess of the anti-hepcidin molecule PRS-080 prior to WCX-TOF MS analysis (**C**); Panels **D/E**, control peptide profile of plasma from patients with juvenile hemochromatosis and iron deficiency anemia, respectively, that lack hepcidin-25. Positions in the spectrum: hepcidin-25^+40^ (internal standard), m/z 2829.4; hepcidin-25, m/z 2789.4; hepcidin-24, m/z 2673.9; hepcidin-22, m/z 2436.1; and hepcidin-20, m/z 2191.8. It should be noted that: i) profiles from IC and nephrology patients both clearly contain the m/z 2673.9 peak at the presumed position of hepcidin-24; ii) this peak disappears completely from the profile after incubation with PRS-080, similar to hepcidin-25/-22; iii) hepcidin-25^+40^ does not disappear from the profile as it was added after the PRS-080 incubation period, which limits complex formation; iv) the intensity of the presumed peak of hepcidin-20 at m/z 2191.8 after PRS-080 incubation decreases but does not disappear completely suggesting that another hepcidin-unrelated peptide is also present at this position; v) the peptide spectra of patient that lack hepcidin-25 also contain a peak at m/z 2191.8 (calculated between 1–2 nM), providing further evidence for the unlikeliness that this peak is solely derived from hepcidin-20.

To further test the applicability of hepcidin-25^+40^ as internal standard, it was used to quantify hepcidin-25 and its isoforms in samples from patients for which hepcidin isoforms were expected by WCX-TOF MS. To this purpose, we used samples from 2 heparin plasma pools that are representative for sample from intensive care and nephrology patients. As shown in Figure 4A and B, the resulting peptide profiles showed peaks at the m/z positions of hepcidin-25 and its expected hepcidin-22 and hepcidin-20 isoforms. Surprisingly, these peptide spectra did also contain a peak at m/z 2673.9 that matches the theoretical mass of hepcidin-24 (assuming that 4 intra-molecular disulphide bridges are present). To estimate the prevalence of this peak among individuals from various patients groups, we investigated samples from our bio-repository. Interestingly, the m/z 2673.9 peak was observed in none of 5 randomly selected healthy controls [18], in 7 out of 10 patients with chronic kidney disease [9], in 7 out of 10 patients on hemodialysis [16] and in 8 out of 10 IC patients (collected for the current study). In the patients (n = 20) with kidney failure (either chronic kidney disease or hemodialysis) we detected the putative hepcidin-24 peak in 3 out of 9 patients with ferritin <200 µg/L and in all 11 with a ferritin>200 µg/L, suggesting a relation with iron status and/or inflammation in these patients. To confirm the identity of the putative hepcidin-24 peptide peak, we took advantage of the fact that pre-incubation of plasma samples with the anti-hepcidin molecule PRS-080 (Anticalin) prevents hepcidin binding to WCX beads due to a picomolar affinity of the Anticalin for hepcidin and thereby strong complex formation [29]. As shown in Figure 4C, hepcidin-24 disappeared, like hepcidin-25 and hepcidin-22, completely from the peptide profile after PRS-080 was added to the same plasma pool from nephrology patients that was used to generate the spectrum of panel B. This identity confirmation shows for the first time that hepcidin-24 can be a naturally occurring hepcidin isoform in the circulation of intensive care and nephrology patients. Another interesting observation concerned the fact that the intensity of the presumed peak of hepcidin-20 at m/z 2191.8 did not completely disappear from the spectrum after PRS-080 incubation. This finding suggests that another hepcidin-unrelated peptide is also present at this position as the *in vitro* affinity of PRS-080 is similar for both hepcidin-20 and hepcidin-25 (our unpublished observations) and hepcidin-25, hepcidin-24 and hepcidin-22 could be completely depleted with the employed excess of Anticalin. The latter idea is corroborated by the peptide profiles that belong to patients with iron deficiency anemia or iron-depleted juvenile hemochromatosis that lack hepcidin-25, but also do contain a detectable peak at position m/z 2191.8 (Figure 4D and E). Thus, it is highly unlikely that the peak that is observed at this position in the IC and nephrology samples is solely derived from the hepcidin-20 isoform and clearly points out that hepcidin isoform analysis on low/medium resolution platforms should be analyzed with great care. It advocates the regular validation of the peptide identity based on immune-depletion/capture on these platforms or MS/MS based peptide profiling of the involved peptide peaks when assessing different patient groups.

### Bioactivity of the Newly Identified Hepcidin-24

**Figure 5:**
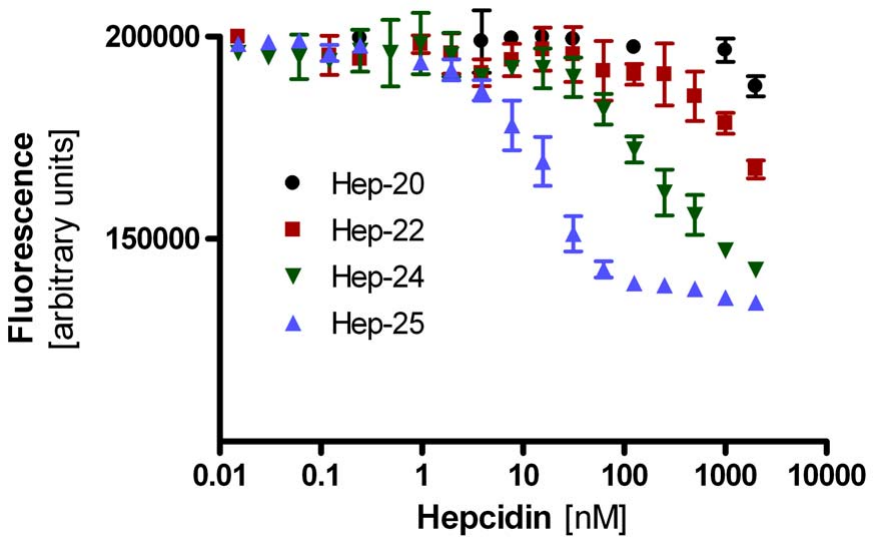
Hepcidin-mediated ferroportin internalization. Different concentrations of synthetic hepcidin-25, -24, -22 and -20 (indicated in nM on the horizontal axis) were added to the growth medium of a stable cell line that expresses green fluorescent protein-fused ferroportin (GFP-FPN). Hepcidin-mediated GFP-FPN internalization and degradation was quantified by measuring cellular fluorescence levels in arbitrary units.

To investigate possible clinical implications of the presence of circulating hepcidin-24 in certain patient groups, we quantified the ability of the newly detected hepcidin isoform to internalize and degrade GFP-fused ferroportin in a cell-based assay [32], [33], relative to that of hepcidin-25, -22 and -20. As illustrated in Figure 5, the EC50 for hepcidin−24 was with 191.5 nM (95% confidence interval 126.8 nM - 289.2 nM) about 10-fold higher than that of hepcidin−25 with 14.7 nM (95% confidence interval 12.2 nM –17.8 nM), indicating a 10-fold lower activity of this newly identified hepcidin isoform. Notably, the activities of the shorter hepcidin-22 and -20 isoforms were at least 10-fold lower than that of hepcidin−24, which is in-line with the previously reported effects of truncation at the amino-terminus [33], [34]. In view of the fact that the hepcidin-24 levels *in vivo* are at least 10-fold lower than those of hepcidin-25 (see Figure 4; data not shown), it is not anticipated that the residual bioactivity of hepcidin-24 contributes significantly to the ferroportin-regulating potential in these individuals with high circulating hepcidin-25/isoform levels.

### Clinical Validation: Comparing IS Hepcidin-24 and Heavy Hepcidin

**Figure 6:**
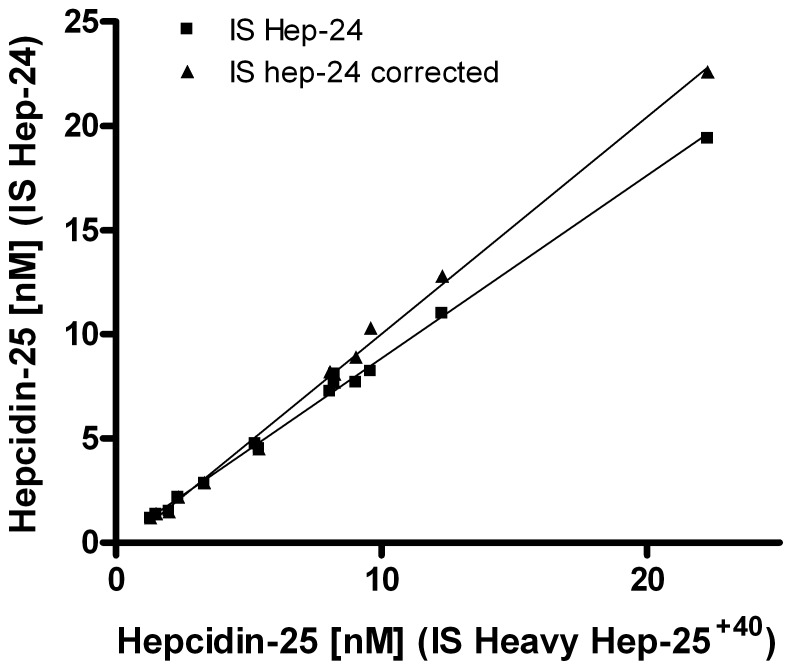
Comparison of hepcidin concentrations obtained by the respective internal standards hepcidin-24 and hepcidin-25^+40^, with and without correction for native hepcidin-24 concentrations. Samples (n = 14) consisted of serum samples from healthy controls (n = 3), heparin plasma from nephrology patients (n = 7), heparin plasma high and low QC pools, serum high and low QC pools. Description of the lines: hepcidin-25 (IS HEP-24), Y = 0.878X+0.059 (R^2^ = 0.9959); hepcidin-25 (IS HEP-24), with hep-24 correction, Y = 1.041X−0.425 (R^2^ = 0.9960).

To validate the clinical use of our updated assay, we measured the hepcidin-25 concentrations in a variety of samples (n = 14) using either the novel hepcidin-25^+40^ isotope or synthetic hepcidin-24 as standard. We observed that values obtained by using the heavy isotope hepcidin-25 were slightly higher in samples that contained hepcidin isoforms than those obtained by our former hepcidin-24 standard, nevertheless the results of both assay had a strong correlation (Figure 6). Interestingly, upon correction of the hepcidin-25 values obtained by using the hepcidin-24 as internal standard, by the concentration of native hepcidin-24 in these samples measured by the use of the hepcidin-25^+40^ standard, both methodologies were nearly identical (Figure 6). This clearly shows that use of the novel hepcidin-25^+40^ internal standard leads to improved quantification in samples that contain hepcidin isoforms. On the other hand, it shows that hepcidin-24 is a convenient surrogate internal standard in samples that do not contain hepcidin isoforms.

### Reproducibility of the Measurement of Hepcidin-25 and its Isoforms

To assess the robustness of the improved hepcidin assay with hepcidin-25^+40^ as internal standard, we determined coefficient of variations (CV’s) by repeated intra-run and inter-run measurements (n = 8) of samples with different concentrations of native hepcidin-25 and its isoforms (Table 2). These data are indicative of a reliable TOF MS assay for assessment of hepcidin-25 and its isoforms. They show higher CV’s for the smaller hepcidin isoforms especially for the inter-run measurements and in the lower concentration ranges at which these smaller isoforms are generally observed.

### Value Assignment: Effect of Vendor of Synthetic Hepcidin-25

**Figure 7:**
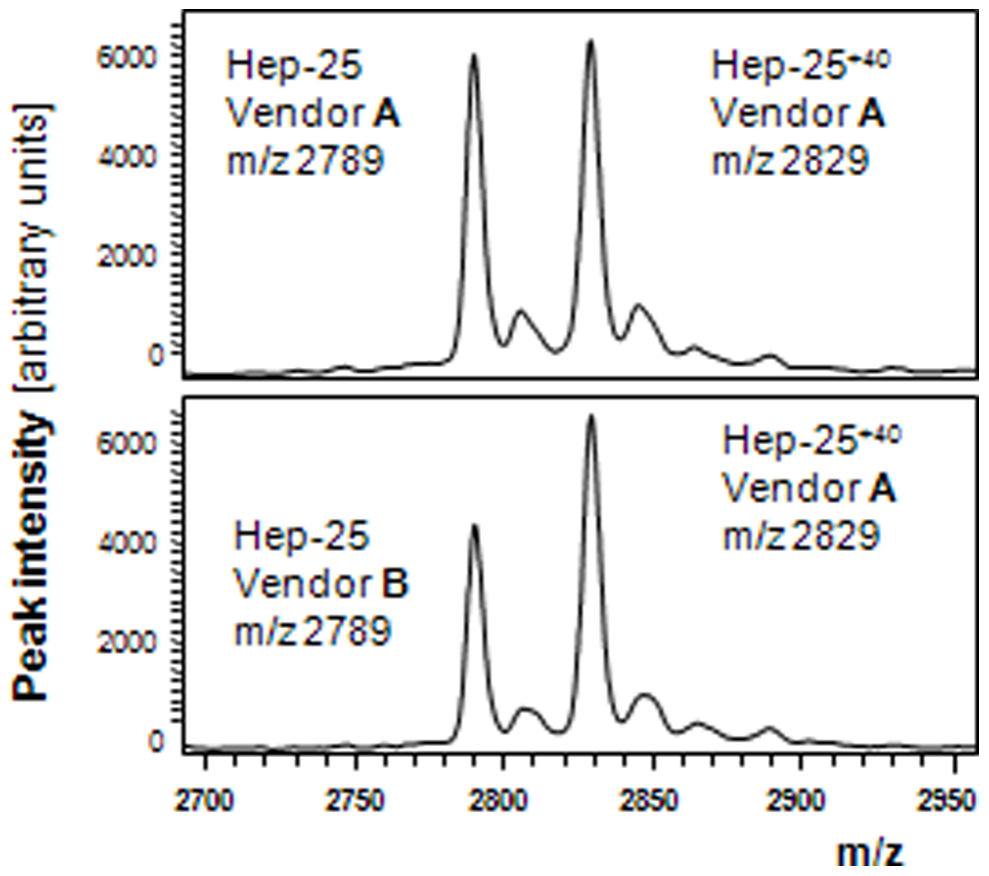
Peak Intensity of synthetic Hepcidin-25 of Peptides International (Vendor A; top panel) and Bachem (Vendor B; bottom panel) spiked to blank serum (to obtain a 10 nM concentration) and measured by WCX-TOF MS, using hepcidin-25^+40^ from Peptides International as internal standard. The theoretical concentrations of hepcidin-25 used in these experiments were adjusted towards 100% peptide content, based on the information provide in the package inserts of the respective Vendors (see Table 1). Hep-25, m/z 2789.4; Hep-25^+40^ (internal standard), m/z 2829.4.

To better understand the cause of different absolute hepcidin concentrations that are measured by different hepcidin assays throughout the world [25], we used the hepcidin-25^+40^ standard to quantify synthetic hepcidin peptides from either Bachem or Peptides International that were spiked in blank serum with 10 nM of synthetic hepcidin-25 (according to the package inserts of the vendors). In the samples spiked with synthetic hepcidin from Peptides International we observed a mean concentration of 9.91 nM (n = 2) hepcidin-25, whereas this was 6.86 nM (n = 2) for the synthetic peptide from Bachem (Figure 7). These findings corroborate previous reported data and indicate that a significant difference in value assignment exists between the hepcidin-25 peptides from these two vendors, even when corrected towards a 100% peptide content of hepcidin-25 [25]. Consequently, if both would be used to quantify hepcidin, than the assay in which the Bachem peptide was used would give about 1.5-fold higher concentrations than when an internal standard from Peptides International was used.

### Value Assignment Assessed by Immuno-depletion with Hepcidin Antagonists

**Figure 8:**
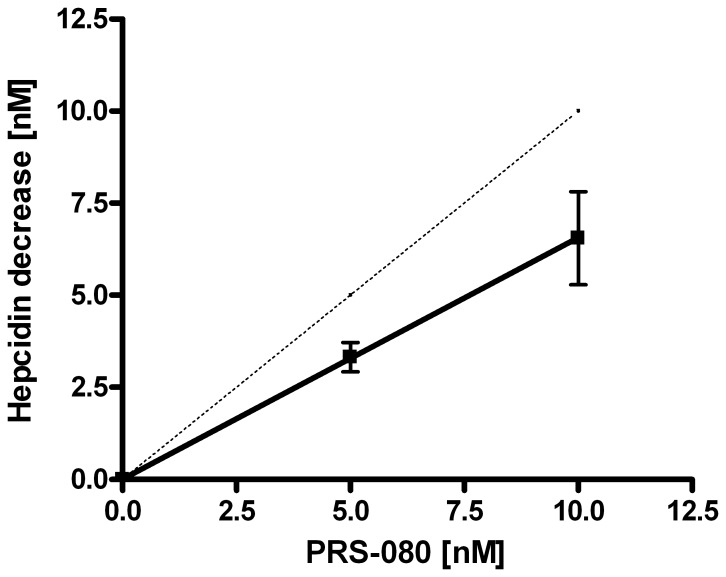
Hepcidin quantification by WCX-TOF MS in human samples that were pre-incubated with 0, 5 or 10 nM of PRS-080 to block binding of hepcidin to the WCX beads. Decrease in hepcidin concentration was determined by measurement of 6 different samples, with total hepcidin concentrations between 10 and 40-080. Dotted line indicates the theoretically expected 1∶1 ratio. Description of the line: Y = 0.655X+0.013 (R^2^ = 0.9373).

To get an impression of the accuracy of the absolute hepcidin concentrations measured by our assay, we aimed to compare the decrease in hepcidin concentration in relation to the addition of known concentrations of the hepcidin-specific Anticalin PRS-080 that blocks binding of hepcidin to WCX beads during the affinity enrichment step of our assay. As shown in Figure 8, we observed a near equimolar decrease in hepcidin upon addition of increasing concentrations of PRS-080 for six separate native samples that did or did not contain hepcidin isoforms with concentrations between 10 nM and 40 nM of total hepcidin (sum of concentrations of hepcidin-25 and isoforms). More specifically, our observations were consistent with a molar ratio PRS-080 to hepcidin-25 of 1.53∶1, suggesting that the absolute concentrations of hepcidin that are measured by our WCX-TOF MS assay are slightly too high assuming that all PRS-080 and hepcidin molecules have a confirmation that enables complex formation. Importantly, these data show that the hepcidin concentrations measured by the WCX-TOF MS assay come near to the real absolute values and are at least in the correct order of magnitude. The fact that we measure slightly higher levels than expected indicates that a minor fraction of the hepcidin-25^+40^ peptide is lost by sticking to pipette tips and/or tubes during preparation of the standard and does not end up in the stock solution of the internal standard, which thus is assigned a somewhat too high concentration. Based on our current experiments we predict that our thus far reported levels should be corrected by a factor 0.65 to approximate the absolute hepcidin levels in a sample. However, it should be emphasized that in biomedical studies the relative differences in hepcidin levels between study groups are more important than the absolute levels and that most currently available hepcidin assays are able to accurately do so, but do not allow inter-assay comparisons [25].

### Effect of Sampling Material and Storage Conditions on Hepcidin-25 and its Isoforms

#### Storage at room temperature

To assess the stability of hepcidin in serum and plasma matrices, we kept samples from intensive care patients (n = 20) and healthy controls (n = 5) at room temperature for 0–7 days followed by WCX-TOF MS analysis using the hepcidin-25^+40^ internal standard. For heparin plasma samples, the results showed that hepcidin-25 gradually decreased at a rate that was highly sample dependent (Table 3, Figure S1A). For the samples at day 1 and day 7, the mean decrease in hepcidin-25 for the 5 controls and 20 intensive care patients was 90% (CV, 8% ) and 55% (CV, 23%), respectively (Table 3).

**Table 3:** Relative change of hepcidin at RT in heparin plasma*.

	Hepcidin level after one day at RT (%)				
	Hep-25	Hep-24	Hep-22	Hep-20	Total-Hep
average	90	120	127	115	98
CV	8	21	14	25	5
+2 SD	103	169	163	171	108
−2 SD	76	71	91	58	87
**n = **	**24**	**15**	**13**	**18**	**11**
	**Hepcidin level after one week at RT (%)**				
	**Hep-25**	**Hep-24**	**Hep-22**	**Hep-20**	**Total-Hep**
average	55	104	150	138	71
CV	23	29	29	38	17
+2 SD	80	164	237	242	94
−2 SD	30	44	62	34	47
**n = **	**24**	**15**	**13**	**18**	**11**

* Relative change in hepcidin-25, -24, -22 and -20 concentrations in heparin plasma samples expressed as percentage of the respective concentrations in fresh samples. Samples were from 19 intensive care (IC) patients and 5 controls and kept for 1 day and 7 days (1 week) at RT. Results <1.0 nM were removed from the calculations, among which are the results of the isoforms of 5 controls. Only results from samples with complete serial measurements are included.

Besides hepcidin-25 we also measured the smaller hepcidin-isoforms (Figure S1B-D, Table 3). In control samples, only the isoform hepcidin-20 emerged in time. However, the quantitative data of the isoform in time were considered not to be reliable since levels remained below 1 nM, and thus were associated with high inter-run CV’s.

**Figure 9:**
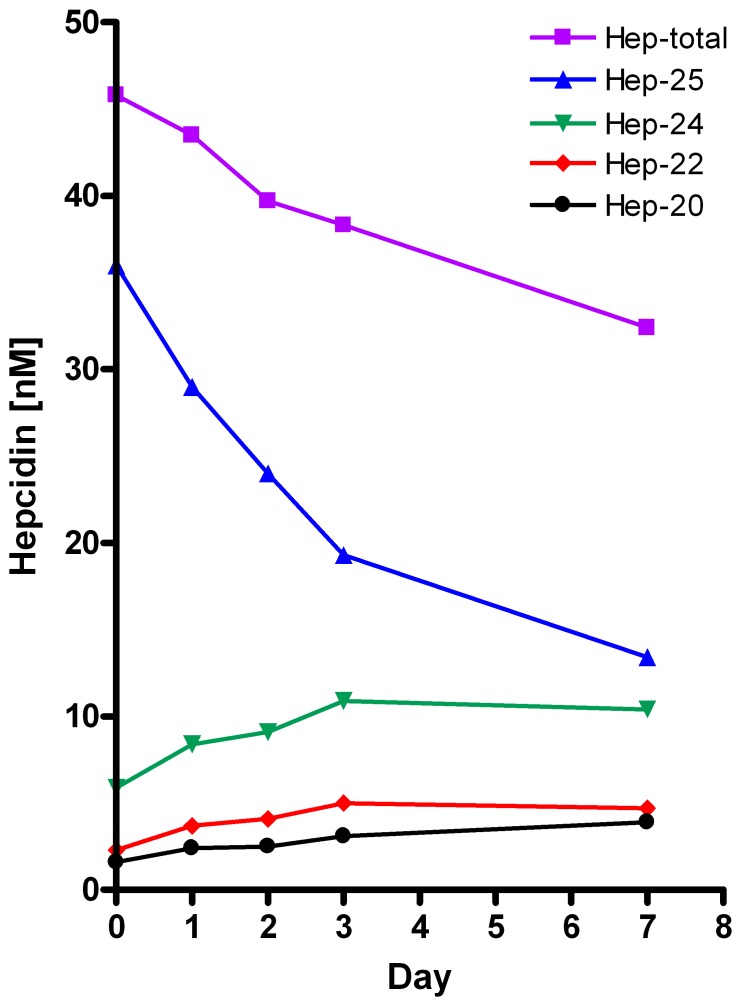
Changes in time at RT of hepcidin-25, 24, 22, and 20 and their sum (total hepcidin) in a representative heparin sample of an IC patient to illustrate that decrease in hepcidin-25 levels is accompanied by an increase in hepcidin-24, -22, and -20 levels. Similar observations for an extended set of samples are shown in Figure S1. Note that the total amount of hepcidin decreases despite the increase in hepcidin isoform levels.

Interestingly, as illustrated by Figure 9, for samples from intensive care patients, the initial decrease in hepcidin-25 was paralleled by an increase in the smaller hepcidin isoforms during this period. For these patients, the respective mean increase of the hepcidin isoforms at day 7 were 116% (31%) for hepcidin-24, 160 (23%) for hepcidin-22, and 164% (41%) for hepcidin-20 (Table S1). However, the total amount of hepcidin decreased to −29% (17%) at day 7 (Table 3), suggesting that hepcidin-isoforms are degraded in smaller pieces that are not detected in our mass-spectrometry setup (Figure 9, Table 3).

For hepcidin-25 similar results were obtained for serum, EDTA plasma and citrate plasma of 5 healthy control subjects (Figure S2). Addition of protease inhibitors to heparin samples of IC patients (n = 10) prevented hepcidin-25 processing to a certain extend but did not block its decrease completely (Table S1).

Thus, since a 0–7 day stay of samples on the laboratory bench of samples with and without hepcidin isoforms results in *ex-vivo* changes in hepcidin-25 and its smaller isoforms, delays in measurements or in aliquoting for storage at lower temperatures should be prevented. These results differ from those of Itkonen et al. [35] who found hepcidin-25 levels of serum from 12 healthy controls to be stable for 1 day at room temperature.

#### Storage at 4°C

In heparin and EDTA plasma samples from intensive care patients (n = 10) and healthy controls (n = 5) and citrate-plasma and serum samples from healthy controls (n = 5) that were kept at 4°C for 0–7 days, concentrations of hepcidin−25, remained stable (Table S2/Figure S2). The same holds for total hepcidin and hepcidin isoforms of the intensive care patients (with the exception for hepcidin-20 in EDTA plasma, see Table S2). However, in these samples we cannot fully exclude small changes in hepcidin isoforms in time due to the relatively high inter-run CV’s of their measurement. In controls concentrations of hepcidin isoforms remained <1 nM in time (data not shown).

Altogether, these data merely agree with the previously reported 6-day stability found for serum in 12 healthy controls [35] and indicate that samples can be shipped at 4°C in case this temperature can be guaranteed during transport of less than 7 days.

#### Storage at −20°C

Hepcidin-25 concentrations in i) serum and heparin plasma, EDTA plasma, and citrate plasma samples of healthy controls (n = 5) stored at −20°C did not change significantly for at least 6 months and ii) EDTA plasma and heparin plasma samples of 10 intensive care patients and 5 controls stored at −20°C remained stable for at least 4 weeks (Tables S3 and S4). In time, concentrations of hepcidin isoforms remained <1 nM for controls (data not shown) and did not change in time for IC patients.

We observed no differences between the various anticoagulants used. We observed, however, a small non significant decrease of hepcidin-25 upon freezing (e.g. for heparin samples at 7 days: total intensive care and control (n = 15): 95% (CV 8%) relative to fresh samples, Table S3). Hepcidin isoforms did not change significantly in time. However, it should be noted that the reproducibility of the measurements of the smaller isoforms at low concentrations is low, especially the measurements of hepcidin-20 in EDTA plasma samples (Table 2, Table S3). Thus, hepcidin measurements by WCX-TOF MS are not influenced by storage at −20°C for at least 4 weeks, except for a possible small decrease in hepcidin-25 upon freezing. These data corroborate previous findings of a stability for serum at last 42 days in healthy controls [35]. Moreover, storage of EDTA plasma samples at −20°C does provide unreliable hepcidin-20 results with WCX-TOF MS.

#### Storage at −80°C

Hepcidin-25 results in QC serum samples measured by our former hepcidin-24 standard remained stable for 2 years at −80°C. Thereafter CV’s increased, and control values regularly were out of range, necessitating re-analyses of the samples (Figure S3). This increase of CV’s of hepcidin-25 measurements are most likely due to increasing noise in the MS profile upon prolonged storage.

Hepcidin-25 results in QC heparin plasma samples stored at −80°C and measured with the heavy hepcidin standard are stable for at least 1.5 years (Figure S3).

Storage of serum, heparin, EDTA and citrate plasma of 5 healthy volunteers results in a small but not significant average decrease of 4–6% in hepcidin-25 concentrations after 6 months (Table S5).

We conclude that hepcidin-25 results are not changed during 2 years of storage at −80°C, except for a possible small decrease upon freezing. However, after 2 years storage at −80°C, hepcidin results become less precise and individual measurements might become less reliable. Average results for a population may not be affected, but existing differences in hepcidin levels between groups, or correlations of hepcidin with other parameters might be more difficult to assess. These data extend previous reports describing hepcidin stability at −80°C for at least 6–8 months [23], [36], [37].

## Concluding Remarks

Our WCX-TOF MS assay has proven to be an accurate and reproducible methodology to quantify hepcidin-25 and provides physiologically relevant results that were published in more than 60 biomedical studies over the last five years (see: www.hepcidinanalysis.com). Here we show that the implementation of the hepcidin-25^+40^ isotope, which replaces hepcidin-24 as a internal standard, further improves the test characteristics of this methodology, especially with respect to samples from patients with hepcidin isoforms. Our data on the stability of hepcidin-25 and its isoforms under various conditions will be helpful in the design of clinical studies. The implementation of the hepcidin-25^+40^ now also allows the identification and quantification of hepcidin-24 as a novel isoform in specific patient groups. Importantly, we have pinpointed critical steps in the handling and preparation of the internal standards in general to provide clues for a more accurate determination of the absolute hepcidin concentration. We strongly believe that different sources and protocols for preparation of hepcidin standards is most strongly contributing to the differences in values between methodologies as observed in the world-wide round robins [25], [26]. The difference in hepcidin results obtained with standards from different vendors suggests issues with value assignment of these materials, while the choice of dissolvent significantly affects the recovery of the reference peptide. Suboptimal materials (lower starting amount) and protocols (lost of standard due to sticking to laboratory plastics) all lead to a lower actual concentration of the hepcidin standard than expected and used to calculate the measured hepcidin concentration. This leads to false high measured hepcidin levels and this observation implies that the assays that report the lowest hepcidin concentration may be most accurate in terms of absolute quantification. Interestingly, in the Round Robins for serum hepcidin our WCX-TOF MS (Method II in [26]; MS1 in Table 2 and onwards in [25]), was among the assays that measured lowest absolute hepcidin concentrations. Our titration experiments with the hepcidin-specific Anticalin PRS-080 seems to indicate that even our optimized protocols do not allow full recovery of the internal standard and that we may still measure about 1.53-fold higher hepcidin levels than actually present in a sample. Nevertheless, the hepcidin-25^+40^ peptide could aid in the desired harmonization of hepcidin throughout the world. As the relative large mass shift of 40 Da makes this isotope suitable for easy-to-use medium resolution linear TOF platforms, but can also be used on high-resolution mass spectrometry platforms as well as immunoassays. Therefore, this peptide is a strong candidate to be introduced as higher order reference materials to validate the different reference peptides that are in use throughout the world. Taken together, our present study aids in the understanding of circulating hepcidin-25 and its isoforms and provide important leads for the further harmonization of plasma hepcidin assays.

## Supporting Information

Figure S1 Changes in concentrations of hepcidin-25 (**A**), hepcidin isoforms (**B, C, D**) in heparin samples from IC patients (n = 19 in black) and healthy controls (n = 5, in red) kept for 0–7 days at room temperature. Only samples with complete serial measurements are included; results <1 nM were excluded. None of the freshly collected samples from healthy control subjects contained hepcidin isoforms levels >1 nM.
(TIF)

Figure S2 Changes in concentrations of hepcidin-25 in heparin, EDTA and citrate plasma as well as serum in samples from the same 5 healthy controls (collected at the same moment) kept for 0–7 days at room temperature (RT) or 4°C.
(TIF)

Figure S3 Control charts of results from HiQC samples measured throughout time. A, serum; B, plasma. QC samples were aliquoted and stored at −80°C, a fresh aliquot was used for each measurement. Deviation starts to increase after 2 years for the serum QC sample, the plasma QC sample is still stable after 1.5 years.
(PDF)

Table S1 Relative change of hepcidin-25, -24, -22, -20 concentrations in heparin samples from 10 intensive care (IC) patients after 1 day (**A**) and 1 week (7 days; **B**) at room temperature (RT) with and without addition of protease inhibitors.
(DOC)

Table S2 Relative change of hepcidin-25, -24, -22 and -20 concentrations in heparin and EDTA plasma samples from 10 intensive care (IC) patients and 5 controls after 1 day (**A**) and after 1 weeks (7 days; **B**) at 4°C. *Results of samples with measured hepcidin <1.0 nM were deleted from the calculations. Samples from controls did not contain hepcidin isoform levels >1.0 nM; #, Measurements of hepcidin-20 in stored EDTA plasma proved unreliable due to the variable and unexplained appearance of an additional peak of similar mass in the WCX-TOF MS profile.
(DOC)

Table S3 Relative change of hepcidin-25, -24, -22 and -20 concentrations in heparin and EDTA plasma samples from 10 intensive care (IC) patients and 5 controls after 1 week (**A**) or 1 month (4 weeks; **B**) at −20°C. *Results of samples with measured hepcidin <1.0 nM were deleted from the calculations. Samples from controls did not contain hepcidin isoform levels >1.0 nM; #, Measurements of hepcidin-20 in stored EDTA plasma proved unreliable due to the variable and unexplained appearance of an additional peak of similar mass in the WCX-TOF MS profile, e.g. for EDTA plasma samples in 7 out of 10 patients, intra-individual changes in hepcidin-20 in time (fresh, week 1, week 4) showed an outlier (defined as 1 out of 3 serial measurements >100% different from other 2 measurements).
(DOC)

Table S4 Relative change of hepcidin-25 concentration in heparin -, EDTA-, and citrate plasma and serum from 5 controls after 1 week (wk), 1 month (mnt), 4 months (mnts) and 6 mnts at −20°C.
(DOC)

Table S5 Relative change of hepcidin-25 concentration in heparin -, EDTA-, and citrate plasma and serum from 5 controls after 6 months at −80°C.
(DOC)
