# Supplementary figures and images for: Improved Mass Spectrometry Assay For Plasma Hepcidin: Detection and Characterization of a Novel Hepcidin Isoform
Source: PLoS One. 2013 Oct 4;8(10):e75518. doi: 10.1371/journal.pone.0075518 (PMC3790851; doi:10.1371/journal.pone.0075518)

**A** control chart HiQC 2011 hepcidin-25

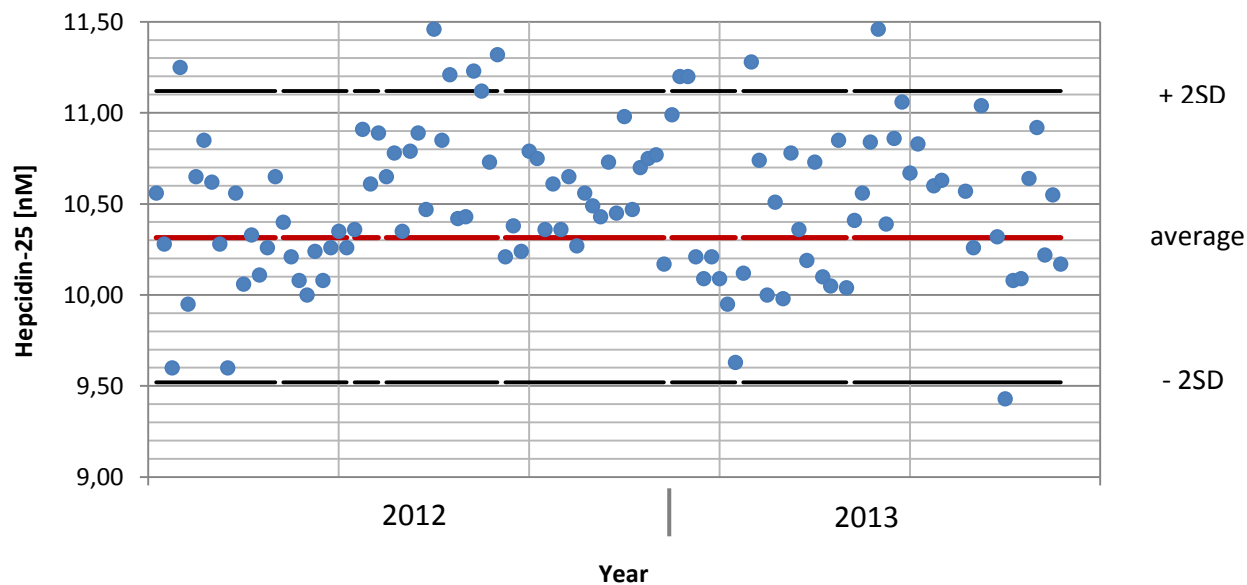

**B** control chart HiQC 2009 hepcidin-25

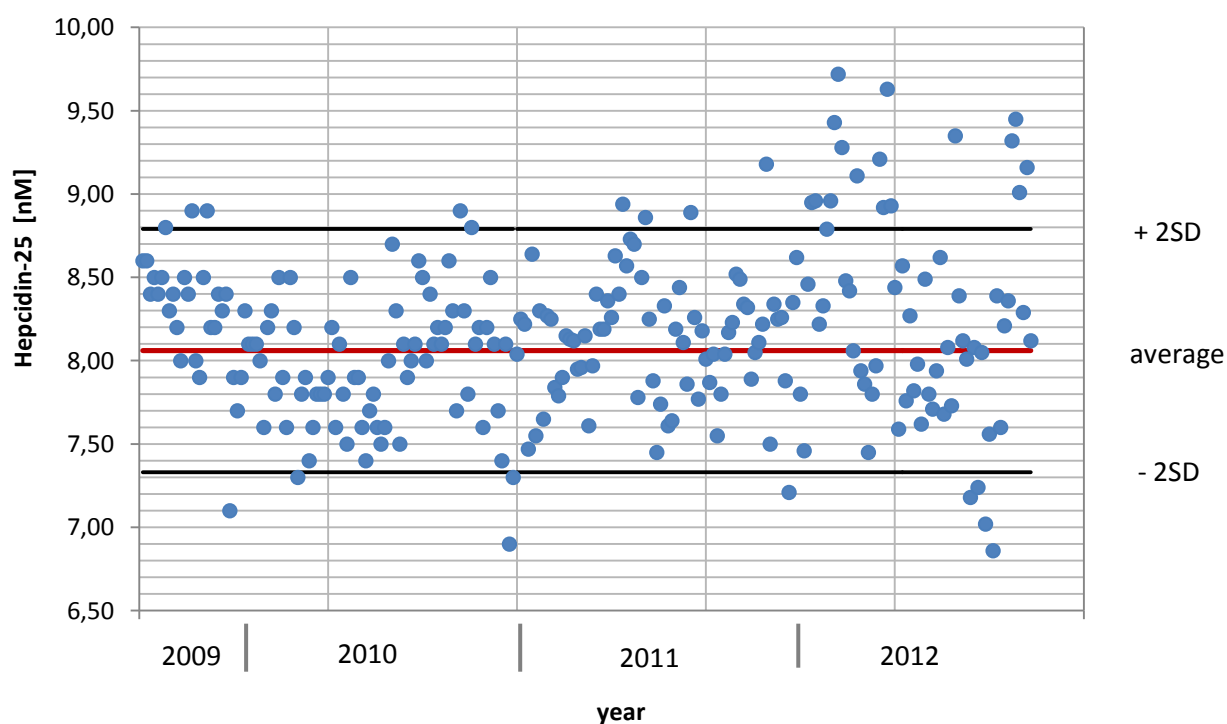

Supplement: Figure S3 — Control charts of results from HiQC samples measured throughout time. A, serum; B, plasma. QC samples were aliquoted and stored at −80°C, a fresh aliquot was used for each measurement. Deviation starts to increase after 2 years for the serum QC sample, the plasma QC sample is still stable after 1.5 years. (PDF) [file pone.0075518.s003.pdf]
