# Supplementary material for: Improved Mass Spectrometry Assay For Plasma Hepcidin: Detection and Characterization of a Novel Hepcidin Isoform
Source: PLoS One. 2013 Oct 4;8(10):e75518. doi: 10.1371/journal.pone.0075518 (PMC3790851; doi:10.1371/journal.pone.0075518)
Supplement: Table S1 — Relative change of hepcidin-25, -24, -22, -20 concentrations in heparin samples from 10 intensive care (IC) patients after 1 day (A) and 1 week (7 days; B) at room temperature (RT) with and without addition of protease inhibitors. (DOC) [file pone.0075518.s004.doc]

**Table S1.** Relative change of hepcidin-25, -24, -22, -20 concentrations in heparin samples from 10 intensive care (IC) patients after 1 day (**A**) and 1 week (7 days; **B**) at room temperature (RT) with and without addition of protease inhibitors. Results <1 nM were excluded. Only results from samples with complete serial measurements are included.

| **A** | **Hepcidin level after one day at RT (%)** | | | | **Hepcidin level after one day at RT (%)** | | | |
| --- | --- | --- | --- | --- | --- | --- | --- | --- |
|  | without protease inhibitors | | | | with protease inhibitors | | | |
|  | Hep-25 | Hep-24 | Hep-22 | Hep-20 | Hep-25 | Hep-24 | Hep-22 | Hep-20 |
| *average* | 89 | 128 | 135 | 141 | 91 | 122 | 105 | 118 |
| *CV* | 13 | 20 | 11 | 11 | 8 | 30 | 4 | 14 |
| *+2 SD* | 112 | 178 | 166 | 172 | 105 | 172 | 114 | 150 |
| *-2 SD* | 67 | 77 | 105 | 110 | 77 | 72 | 97 | 85 |
| **n =** | **10** | **8** | **7** | **8** | **10** | **8** | **7** | **8** |
|  |  | | | |  | | | |
| **B** | **Hepcidin level after one week at RT (%)** | | | | **Hepcidin level after one week at RT (%)** | | | |
|  | without protease inhibitors | | | | with protease inhibitors | | | |
|  | Hep-25 | Hep-24 | Hep-22 | Hep-20 | Hep-25 | Hep-24 | Hep-22 | Hep-20 |
| *average* | 48 | 116 | 160 | 164 | 69 | 133 | 117 | 114 |
| *CV* | 30 | 31 | 23 | 41 | 25 | 35 | 9 | 25 |
| *+2 SD* | 77 | 188 | 234 | 298 | 103 | 227 | 138 | 170 |
| *-2 SD* | 19 | 44 | 86 | 30 | 35 | 40 | 95 | 57 |
| **n =** | **10** | **8** | **7** | **8** | **10** | **8** | **7** | **8** |
