# Supplementary material for: Improved Mass Spectrometry Assay For Plasma Hepcidin: Detection and Characterization of a Novel Hepcidin Isoform
Source: PLoS One. 2013 Oct 4;8(10):e75518. doi: 10.1371/journal.pone.0075518 (PMC3790851; doi:10.1371/journal.pone.0075518)
Supplement: Table S2 — Relative change of hepcidin-25, -24, -22 and -20 concentrations in heparin and EDTA plasma samples from 10 intensive care (IC) patients and 5 controls after 1 day (A) and after 1 weeks (7 days; B) at 4°C. *Results of samples with measured hepcidin <1.0 nM were deleted from the calculations. Samples from controls did not contain hepcidin isoform levels >1.0 nM; #, Measurements of hepcidin-20 in stored EDTA plasma proved unreliable due to the variable and unexplained appearance of an additional peak of similar mass in the WCX-TOF MS profile. (DOC) [file pone.0075518.s005.doc]

**Table S2**. Relative change of hepcidin-25, -24, -22 and -20 concentrations in heparin and EDTA plasma samples from 10 intensive care (IC) patients and 5 controls after 1 day (**A**) and after 1 week (7 days; **B**) at 4 °C.*

| **A** | **Hepcidin level after one day at 4°C (%)** | | | | | | | |
| --- | --- | --- | --- | --- | --- | --- | --- | --- |
|  | Heparin plasma | | | | EDTA plasma | | | |
|  | Hep-25 | Hep-24 | Hep-22 | Hep-20 | Hep-25 | Hep-24 | Hep-22 | Hep-20 |
| average | 99 | 99 | 104 | 93 | 101 | 103 | 104 | 94 |
| CV | 7 | 5 | 13 | 20 | 11 | 6 | 11 | 22 |
| +2 SD | 113 | 107 | 131 | 129 | 124 | 115 | 127 | 135 |
| -2 SD | 85 | 90 | 77 | 56 | 78 | 90 | 81 | 53 |
| **n =** | **15** | **7** | **6** | **10** | **15** | **8** | **6** | **9** |
|  |  | | | | | | | |
| **B** | **Hepcidin level after one week at 4°C (%)** | | | | | | | |
|  | Heparin plasma | | | | EDTA plasma | | | |
|  | Hep-25 | Hep-24 | Hep-22 | Hep-20 | Hep-25 | Hep-24 | Hep-22 | Hep-20^#^ |
| average | 99 | 106 | 96 | 114 | 101 | 113 | 106 | 165 |
| CV | 5 | 8 | 8 | 21 | 9 | 8 | 18 | 21 |
| +2 SD | 109 | 123 | 111 | 162 | 120 | 131 | 144 | 234 |
| -2 SD | 89 | 89 | 81 | 66 | 82 | 95 | 67 | 96 |
| **n =** | **15** | **7** | **6** | **10** | **15** | **8** | **6** | **9** |

*Results of samples with measured hepcidin < 1.0 nM were deleted from the calculations. Samples from controls did not contain hepcidin isoform levels > 1.0 nM; #, Measurements of hepcidin-20 in stored EDTA plasma proved unreliable due to the variable and unexplained appearance of an additional peak of similar mass in the WCX-TOF MS profile.
