# Supplementary material for: Improved Mass Spectrometry Assay For Plasma Hepcidin: Detection and Characterization of a Novel Hepcidin Isoform
Source: PLoS One. 2013 Oct 4;8(10):e75518. doi: 10.1371/journal.pone.0075518 (PMC3790851; doi:10.1371/journal.pone.0075518)
Supplement: Table S3 — Relative change of hepcidin-25, -24, -22 and -20 concentrations in heparin and EDTA plasma samples from 10 intensive care (IC) patients and 5 controls after 1 week (A) or 1 month (4 weeks; B) at −20°C. *Results of samples with measured hepcidin <1.0 nM were deleted from the calculations. Samples from controls did not contain hepcidin isoform levels >1.0 nM; #, Measurements of hepcidin-20 in stored EDTA plasma proved unreliable due to the variable and unexplained appearance of an additional peak of similar mass in the WCX-TOF MS profile, e.g. for EDTA plasma samples in 7 out of 10 patients, intra-individual changes in hepcidin-20 in time (fresh, week 1, week 4) showed an outlier (defined as 1 out of 3 serial measurements >100% different from other 2 measurements). (DOC) [file pone.0075518.s006.doc]

**Table S3**: Relative change of hepcidin-25, 24, 22 and 20 concentrations in heparin and EDTA plasma samples from 10 intensive care (IC) patients and 5 controls after 1 week (**A**) or 1 month (4 weeks; **B**) at -20°C.*

| **A** | **Hepcidin level after one week at -20°C (%)** | | | | | | | |
| --- | --- | --- | --- | --- | --- | --- | --- | --- |
|  | Heparin plasma | | | | EDTA plasma | | | |
|  | Hep-25 | Hep-24 | Hep-22 | Hep-20 | Hep-25 | Hep-24 | Hep-22 | Hep-20^#^ |
| average | 95 | 96 | 87 | 102 | 96 | 96 | 98 | 183 |
| CV | 8 | 11 | 8 | 22 | 11 | 12 | 11 | 28 |
| +2 SD | 110 | 117 | 100 | 147 | 117 | 118 | 120 | 284 |
| -2 SD | 79 | 75 | 73 | 56 | 75 | 71 | 77 | 82 |
| **n =** | **15** | **7** | **6** | **9** | **15** | **8** | **6** | **9** |
|  |  | | | | | | | |
| **B** | **Hepcidin level after one month at -20°C (%)** | | | | | | | |
|  | Heparin plasma | | | | EDTA plasma | | | |
|  | Hep-25 | Hep-24 | Hep-22 | Hep-20 | Hep-25 | Hep-24 | Hep-22 | Hep-20^#^ |
| average | 97 | 114 | 89 | 102 | 99 | 91 | 96 | 210 |
| CV | 29 | 29 | 13 | 25 | 9 | 11 | 19 | 40 |
| +2 SD | 153 | 181 | 112 | 153 | 116 | 111 | 132 | 378 |
| -2 SD | 40 | 47 | 65 | 52 | 81 | 71 | 60 | 41 |
| **n =** | **15** | **8** | **6** | **9** | **15** | **8** | **6** | **9** |

*Results of samples with measured hepcidin < 1.0 nM were deleted from the calculations. Samples from controls did not contain hepcidin isoform levels > 1.0 nM;

#, Measurements of hepcidin-20 in stored EDTA plasma proved unreliable due to the variable and unexplained appearance of an additional peak of similar mass in the WCX-TOF MS profile, e.g. for EDTA plasma samples in 7 out of 10 patients, intra-individual changes in hepcidin-20 in time (fresh, week 1, week 4) showed an outlier (defined as 1 out of 3 serial measurements > 100 % different from other 2 measurements).
