# Supplementary material for: Improved Mass Spectrometry Assay For Plasma Hepcidin: Detection and Characterization of a Novel Hepcidin Isoform
Source: PLoS One. 2013 Oct 4;8(10):e75518. doi: 10.1371/journal.pone.0075518 (PMC3790851; doi:10.1371/journal.pone.0075518)
Supplement: Table S4 — Relative change of hepcidin-25 concentration in heparin -, EDTA-, and citrate plasma and serum from 5 controls after 1 week (wk), 1 month (mnt), 4 months (mnts) and 6 mnts at −20°C. (DOC) [file pone.0075518.s007.doc]

**Table S4**: Relative change of hepcidin-25 concentration in heparin -, EDTA-, and citrate plasma and serum from 5 controls after 1 week (wk), 1 month (mnt), 4 months (mnts) and 6 mnts at -20°C.

|  | **Hepcidin level after storage at -20°C (%)** | | | | | | | |
| --- | --- | --- | --- | --- | --- | --- | --- | --- |
|  | Heparin plasma | | | | EDTA plasma | | | |
|  | 1 wk | 1 mnt | 4 mnts | 6 mnts | 1 wk | 1 mnt | 4 mnts | 6 mnts |
| average | 96 | 92 | 100 | 83 | 89 | 94 | 92 | 88 |
| CV | 6 | 9 | 4 | 19 | 9 | 9 | 9 | 12 |
| +2 SD | 108 | 108 | 108 | 115 | 105 | 111 | 108 | 109 |
| -2 SD | 84 | 75 | 92 | 52 | 73 | 76 | 75 | 67 |
|  |  | | | |  | | | |
|  | Citrate plasma | | | | Serum | | | |
|  | 1 wk | 1 mnt | 4 mnts | 6 mnts | 1 wk | 1 mnt | 4 mnts | 6 mnts |
| average | 97 | 95 | 102 | 97 | 97 | 96 | 98 | 99 |
| CV | 5 | 7 | 4 | 3 | 5 | 3 | 4 | 10 |
| +2 SD | 107 | 108 | 110 | 102 | 107 | 101 | 105 | 119 |
| -2 SD | 87 | 83 | 94 | 92 | 86 | 91 | 90 | 80 |
