# Supplementary material for: Improved Mass Spectrometry Assay For Plasma Hepcidin: Detection and Characterization of a Novel Hepcidin Isoform
Source: PLoS One. 2013 Oct 4;8(10):e75518. doi: 10.1371/journal.pone.0075518 (PMC3790851; doi:10.1371/journal.pone.0075518)
Supplement: Table S5 — Relative change of hepcidin-25 concentration in heparin -, EDTA-, and citrate plasma and serum from 5 controls after 6 months at −80°C. (DOC) [file pone.0075518.s008.doc]

**Table S5**: Relative change of hepcidin-25 concentration in heparin -, EDTA-, and citrate plasma and serum from 5 controls after 6 months at -80°C.

|  | **Hepcidin level after storage at -80°C (%)** | | | |
| --- | --- | --- | --- | --- |
|  | Heparin plasma | EDTA plasma | Citrate plasma | Serum |
| average | 95 | 96 | 96 | 94 |
| CV | 2 | 5 | 5 | 4 |
| +2 SD | 99 | 105 | 106 | 101 |
| -2 SD | 92 | 87 | 87 | 87 |
